# Overexpression of Circular RNA circTTN Promotes Ferroptosis in Sepsis‐Induced Cardiomyopathy

**DOI:** 10.1111/jcmm.71271

**Published:** 2026-07-03

**Authors:** Qian Lei, Danyang Zhao, Zhen Wei, Xue Li, Kunjiao Xue, Tingting Zhang, Juanjuan Lyu

**Affiliations:** ^1^ Department of Anesthesiology Sichuan Academy of Medical Sciences and Sichuan Provincial People's Hospital, School of Medicine, University of Electronic Science and Technology of China Chengdu China; ^2^ Department of Pediatrics West China Second University Hospital, Sichuan University Chengdu China; ^3^ Key Laboratory of Birth Defects and Related Diseases of Women and Children, Ministry of Education West China Second University Hospital, Sichuan University Chengdu China

**Keywords:** circRNA, ferroptosis, heat shock protein, septic cardiomyopathy

## Abstract

Sepsis‐induced cardiomyopathy (SCM) is one of the common complications caused by sepsis. Despite the higher mortality rate associated with SCM, the pathogenesis of SCM is poorly understood. The study of circular RNA (circRNA) in a variety of diseases has accelerated over recent decades. However, the function of circRNAs in SCM is rarely reported. Here, we found that the circRNA from the TTN gene (circTTN) was increased in the SCM mouse and cell models. Modulation of circTTN affected the characteristics of SCM both in mouse and cell models. The expression of ferroptosis regulators, GPX4 and SLC7A11, in SCM models changes inversely with the level of circTTN. Function analysis revealed that circTTN promotes ferroptosis through binding to heat shock protein family A (HSPA5) protein. This finding may promote knowledge about the pathogenesis of SCM and may be helpful for targeted therapy based on circTTN in the future.

## 1 Introduction

Sepsis is defined as the dysfunction of a system or organ due to the body's dysregulated response to inflammatory processes [1, 2]. If not identified promptly and addressed appropriately, it can result in shock, multiple organ failure and potentially fatal outcomes [3, 4]. With the characteristics of high morbidity and mortality, it has become the first major cause of death in acute and critical patients [5, 6]. Sepsis cardiomyopathy (SCM) represents a prevalent complication arising from sepsis, characterised by reversible left ventricular systolic dysfunction [7, 8, 9, 10]. This condition manifests as diminished cardiac output, shock, and left ventricular dilation, which may occur with or without accompanying right ventricular failure [11]. Consequently, SCM significantly elevates the mortality rate among patients suffering from sepsis, reaching approximately 70% to 90% [12, 13]. It is one of the leading causes of death in patients with sepsis [8, 14]. Currently, there is no explanation for the pathogenesis of septic cardiomyopathy. However, a thorough investigation into its mechanisms and the identification of potential therapeutic targets may decrease mortality rates in patients and provide promising advancements in clinical practice [7, 15]. Several new phenotypes have been reported to be associated with SCM, such as ferroptosis [16, 17]. Ferroptosis is a novel form of regulated cell death characterised by iron‐dependent lipid peroxidation and the accumulation of reactive oxygen species (ROS) [18, 19, 20]. Several reports have revealed the importance of ferroptosis underlying SCM [16, 21, 22, 23]. However, more work is needed to further elucidate the mechanisms underlying ferroptosis in SCM and to develop targeted therapies that can effectively treat this deadly condition.

Circular RNA (circRNA) represents a unique form of RNA characterised by a continuous closed‐loop structure, predominantly located in the cytoplasm [24, 25]. The inherent single‐stranded closed circular configuration of circRNAs contributes to their enhanced stability and evolutionary conservation when compared to linear RNAs [24, 26, 27]. These circRNAs are synthesised through a process known as back‐splicing, which involves the exons of protein‐coding genes [27]. The functional repertoire of circRNAs encompasses several roles: (a) serving as a molecular sponge for microRNAs (miRNAs), (b) engaging in interactions with various proteins, (c) modulating protein translation, and (d) influencing the transcriptional activity of linear RNAs [28, 29, 30, 31, 32]. Notably, certain circRNAs can fulfil multiple functions simultaneously [24, 33, 34]. Over recent decades, the investigation into circRNAs has gained significant momentum, leading to a substantial body of literature elucidating their roles in a wide array of human diseases [35, 36]. Nevertheless, there remains a deficit in the exploration of circRNA functions specifically in the context of SCM.

In the present study, we discovered that the level of circRNA from the Titin (TTN) gene, named circTTN, was higher in the SCM mouse and cell models. Function analysis revealed that circTTN promotes ferroptosis in SCM through binding to heat shock protein 70 kDa protein 5 (HSPA5). This finding may promote knowledge about the pathogenesis of SCM and may be helpful for targeted therapy based on circTTN in the future.

## 2 Material and Methods

### 2.1 Animal Model

Male C57BL/6 mice, weighing between 23 and 27 g and aged 8 to 10 weeks, were sourced from the Institute of Laboratory Animals of Sichuan Academy of Medical Sciences. The mice underwent a one‐week acclimatisation period in a controlled environment, during which they had unrestricted access to tap water and a standard commercial diet. Sepsis was induced in mice by cecum ligation and puncture (CLP) as described previously. In brief, mice were subjected to anaesthesia, followed by the ligation of the cecum and the creation of a puncture wound utilising an 18‐gauge needle. In the case of sham operations, sham‐operated mice underwent laparotomy without ligation and puncture.

### 2.2 Echocardiography

Echocardiographic assessments were conducted on the day prior to the surgical intervention and 12 h following CLP. The animals were anaesthetised with 1.5% isoflurane, while their body temperature was consistently maintained at 37.0°C ± 0.2°C. Transthoracic two‐dimensional (2D) and M‐mode echocardiography were executed employing the Vevo 2100 system (FUJIFILM VisualSonics, Toronto, ON, Canada).

### 2.3 Biochemistry Detection

The measurement of mouse NT‐proBNP levels was conducted using a specific kit from Westang (Cat. No: F15151). Additionally, mouse CK‐MB levels were evaluated using a kit provided by Cloud‐Clone Corp. (Cat. No: MEA479Mu). The detection of intracellular reactive oxygen species (ROS) was performed utilising a kit from Beyotime (Cat. No: S0033). Furthermore, intracellular levels of reduced/oxidised glutathione ratio (GSH/GSSG) were assessed with another kit from Beyotime (Cat. No: S0053).

### 2.4 RNA‐Seq Analysis and Sanger Sequencing

Total RNA was extracted from the samples, and ribosome‐depleted RNA was fragmented for first‐ and second‐strand cDNA synthesis using random hexamer primers. Amplification was performed via PCR. A purification and screening process was conducted to identify an appropriately sized library. Ultimately, sequencing of the library was executed using the HiSeq platform. A threshold was established, defining a Fold‐change of ≥ 1.5 or ≤ −1.5 as the cut‐off value. The analysis focused on the significant differential expression of circRNA.

### 2.5 Quantitative Real‐Time Polymerase Chain Reaction (qRT‐PCR)

Total RNA was isolated from both tissue samples and cells utilising TRIzol reagent (Life Technologies). Subsequent to the extraction, the total RNA underwent reverse transcription to generate complementary DNA (cDNA) utilising specific primers as per the instructions of the RT‐PCR kit. qRT‐PCR was conducted employing qPCR SYBR Green Master Mix. The specific primers utilised in the experiments are detailed as follows: forward: 5′‐TGTGTCCGTCGTGGATCTGA‐3′ and reverse: 5′‐TTGCTGTTGAAGTCGCAGGAG‐3′ for GAPDH; forward: 5′‐CGAGGAATGGG AGGAGGACT‐3′ and reverse: 5′‐GCTTCTTGCTTCGCCTCTTG −3′ for circTTN.

### 2.6 Cell Culture and Vector Construction

The HL‐1 cell line was obtained from the ATCC in Manassas, Virginia, USA, and cultured in DMEM medium. The cells were grown in a humidified incubator at 37°C with 5% CO_2_. The circRNA was synthesised by amplifying the mouse TTN sequence utilising the PrimerSTAR Max DNA Polymerase Mix (Takara). PCR products were subsequently cloned into the pCDNA3.1 (+)‐IRES‐EGFP vector, leading to the successful construction of the vector.

### 2.7 RNAi for circTTN

To knock down circTTN in a cell and mouse model, we designed 3 siRNAs for circTTN; the sequence of these siRNAs are as follows: si‐circTTN‐1: sense: 5′‐GGAGGUUCAUGAAGUGUCUUU‐3′; anti‐sense: 5′‐A GACACUUCAUGAA CCUCCUU‐3′; si‐circTTN‐2: sense: 5′‐UGAAGUGUCUAUUCAGUAAUU‐3′; anti‐sense: 5′‐UUACUGAAUAGACACUUCAUU‐3′; si‐circTTN‐3: sense: 5′‐UGAAGCCUCCUGAUAUUCCUU‐3′; anti‐sense: 5′‐GGAAUAUCAGGAGGC UUCAUU‐3′; si‐NC: sense: 5′‐UUCUCCGAACGUGUCACGUUU‐3′; anti‐sense: 5′‐ACGUGACACGUUCGGAGAAUU‐3′.

### 2.8 Cell Transfection

The HL‐1 cell line was seeded onto two six‐well plates, which were subsequently assigned at random to the blank group, negative control group, and overexpression group. At a confluence of 70%–90%, the plasmids were combined with Lipofectamine 2000 in a ratio of 3 μg plasmid to 4 μL Lipofectamine and introduced into the wells containing serum‐free medium. The plates were then incubated at 37°C in an atmosphere containing 5% CO2. Following a culture period of 5–6 h, the transfection medium was discarded and replaced with complete medium. Samples were harvested 48 h post‐transfection. The success of cell transfection was confirmed through PCR analysis. Furthermore, the extracted RNA was assessed using a NanoDrop ND2000 spectrophotometer and analysed via 1% agarose gel electrophoresis.

### 2.9 Cell Counting Kit‐8 (CCK‐8) Assay

A total of four 96‐well plates were inoculated with 100 μL of a cell suspension at a concentration of 1 × 10^4^ cells/mL in each well, 48 h post‐transfection. For each experimental group, three technical replicates were established. The cells were maintained under standard culture conditions, and 10 μL of CCK‐8 solution was introduced into each well at 24, 48, and 72 h post‐inoculation. Subsequently, the plates were incubated for 2 h at 37°C, after which the absorbance was measured at 450 nm.

### 2.10 Cytoplasmic Nuclear Separation

A kit for cytoplasmic nuclear separation was used in this study (Beyotime, Cat. No: P0027). The cytosolic and nuclear components in the cells were separated according to the instructions for operation.

### 2.11 RNA FISH Detection

An anti‐sense probe was designed, and FISH detection was performed in HL‐1 cells. First, the cells are fixed and permeabilised. The anti‐sense probe and NC probe were incubated; hybridisation experiments were carried out overnight at 37°C. After hybridisation, slides were detected with confocal microscopy. All reagents were prepared under RNase‐free conditions.

### 2.12 RNA Pull Down

The strips were cut out and placed into the EP tube. Milli‐Q water was added, and the sample was washed for 1 min. The strips were centrifuged, the supernatant removed, and the pellet washed twice. 50% MeOH/50 mM NH_4_HCO_3_ was then added to decolourise for 30 min and incubated at 37°C. Centrifugation was repeated, and the supernatant was again removed. 100% ACN was then added and stirred for 30 s. The liquid part was then removed when the colloidal particles turned white. A 25 mM DTT/50 mM NH_4_HCO_3_ solution was then added to the dry colloidal particles, reacted at 56°C for 30 min, and the DTT was removed by sucking out. A 55 mM IAA/50 mM NH_4_HCO_3_ solution was then added and reacted in the dark at room temperature for 30 min. IAA was then removed, and Milli‐Q water was added to wash 3 times. A 100% ACN solution was used to dehydrate the colloidal granules till white. Trypsin was diluted with 25 mM NH_4_HCO_3_ to 20 ng/μL. An appropriate amount of trypsin was then added to each tube and put on an ice bath for 30 min. A 25 mM NH_4_HCO_3_ solution was then added to cover the colloidal particles and put into a 37°C incubator for enzymatic digestion overnight. The whole EP tube box was placed into an ultrasonic instrument for 15–20 min, and the peptide was sucked out into a new EP tube.

### 2.13 Mass Spectrometry

Peptides were dissolved in 0.1% formic acid and 2% acetonitrile, then centrifuged at 4°C and 13,200 rpm for 20 min. The supernatant was analysed by mass spectrometry. Isolated peptides were analysed in real‐time using a Thermo Scientific QExactive mass spectrometer, and the resulting data were converted to MGF format via MMFile Conversion software. This MGF file was then utilised to conduct a search against the UniProt database via MASCOT (http://www.matrixscience.com/). The reference database utilised for this study was *Homo sapiens* , accessible at: https://www.uniprot.org/taxonomy/9606.

### 2.14 circRNA Precipitation (circRIP)

Interaction of circTTN with HSPA5 was detected by circRIP. First, HSPA5 was cloned with 3 × Flag fused with HSPA5.3 × Flag‐HSPA5 was overexpressed in HL‐1 cells. circRIP was performed by anti‐Flag antibody; captured RNA was detected by qRT‐PCR. All reagents were prepared under RNase‐free conditions.

### 2.15 Western Blot

Proteins were extracted from cells with reagents, then each fraction underwent SDS‐PAGE and was transferred to a PVDF membrane. Primary antibodies for GAPDH (1:6000, M20006S, Abmaret), H4 (1:800, PTM‐1009, PTM Bio), GPX4 (1:600, 67,763–1‐IG, Proteintech), SLC7A11 (1:600, 26,864–1‐AP, Proteintech), β‐Actin (1:40000, 66,009–1‐Ig, Proteintech), and HSPA5 (1:8000, A23453, ABClonal) were incubated overnight at 4°C. Goat Anti‐Rabbit IgG (1:2000, SA00001‐2, Proteintech) or Goat Anti‐Mouse IgG (1:3000, M21001, ABmart) was used at room temperature for 1 h. Protein bands were detected using enhanced chemiluminescence, with validation through at least three Western blot experiments.

### 2.16 Statistical Analysis

Experimental data were analysed using SPSS 21.0 software (IBM, Armonk, NY, USA), with results expressed as mean ± standard deviation. The means of two independent samples were compared using the *t*‐test, and for multiple groups, one‐way ANOVA with the LSD method was used. A *p*‐value of less than 0.05 was deemed statistically significant.

## 3 Results

### 3.1 circTTN Expression Is Elevated in SCM

**FIGURE 1:**
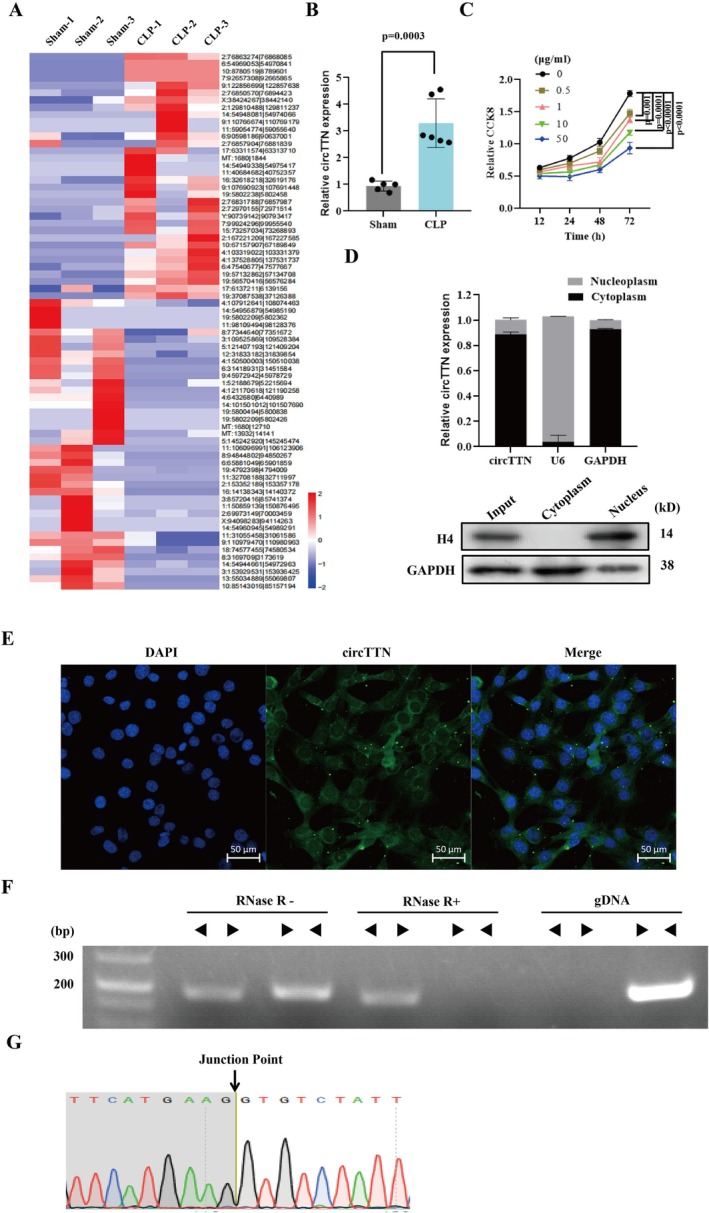
circTTN is elevated in the CLP septicemic cardiomyopathy model. (A) High‐throughput transcriptome detection in 3 CLP model mouse heart tissue samples, heatmap shows the different expressed of circRNAs; (B) RT‐qPCR detection of circTTN in CLP model mouse heart tissue samples (5 sham mice and 6 CLP mice respectively); (C) HL‐1 cell treated with different concentration of LPS for different time periods, CCK8 detection the cell proliferation; (D) Separate the cytoplasm and nuclear components, qPCR detection of circTTN; (E) FISH detection of circTTN in HL‐1 cell; (F) RT‐PCR detection of the circTTN and linear TTN to verify the circular formation of circTTN; (G) Sanger sequencing of circTTN junction point. Three biological repeats were performed for all qPCR detections. Student *t*‐test was used for two‐group comparison, *p < 0.05, **p < 0.05, ***p < 0.001.

In the mouse model of SCM, myocardial samples were collected, and circRNA expression levels were analysed by high‐throughput transcriptome sequencing. It was found that circRNA derived from the TTN gene (2:76863274|76,868,085 in the heatmap) was significantly increased in the SCM myocardial tissue (Figure 1A). Further qPCR detection showed that circTTN was significantly higher in the CLP group than in the sham group (Figure 1B). Lipopolysaccharide (LPS) treated cardiomyocytes is a common septic cardiomyopathy in vitro cell model [37]. LPS inhibits HL‐1 cell proliferation in a dose‐ and time‐dependent manner (Figure 1C). The subcellular localisation of circTTN detected by qPCR after cytoplasmic nuclear separation showed that circTTN was mainly located in the cytoplasm (Figure 1D). This phenomenon was further verified by FISH (Figure 1E). CircTTN is resistant to RNase R digestion (Figure 1F), and Sanger sequencing at potential junction points showed that circTTN was consistent with the expected sequence (Figure 1G).

### 3.2 LPS Induce Inflammatory Response and an Increase of circTTN in the SCM Cell Model

**FIGURE 2:**
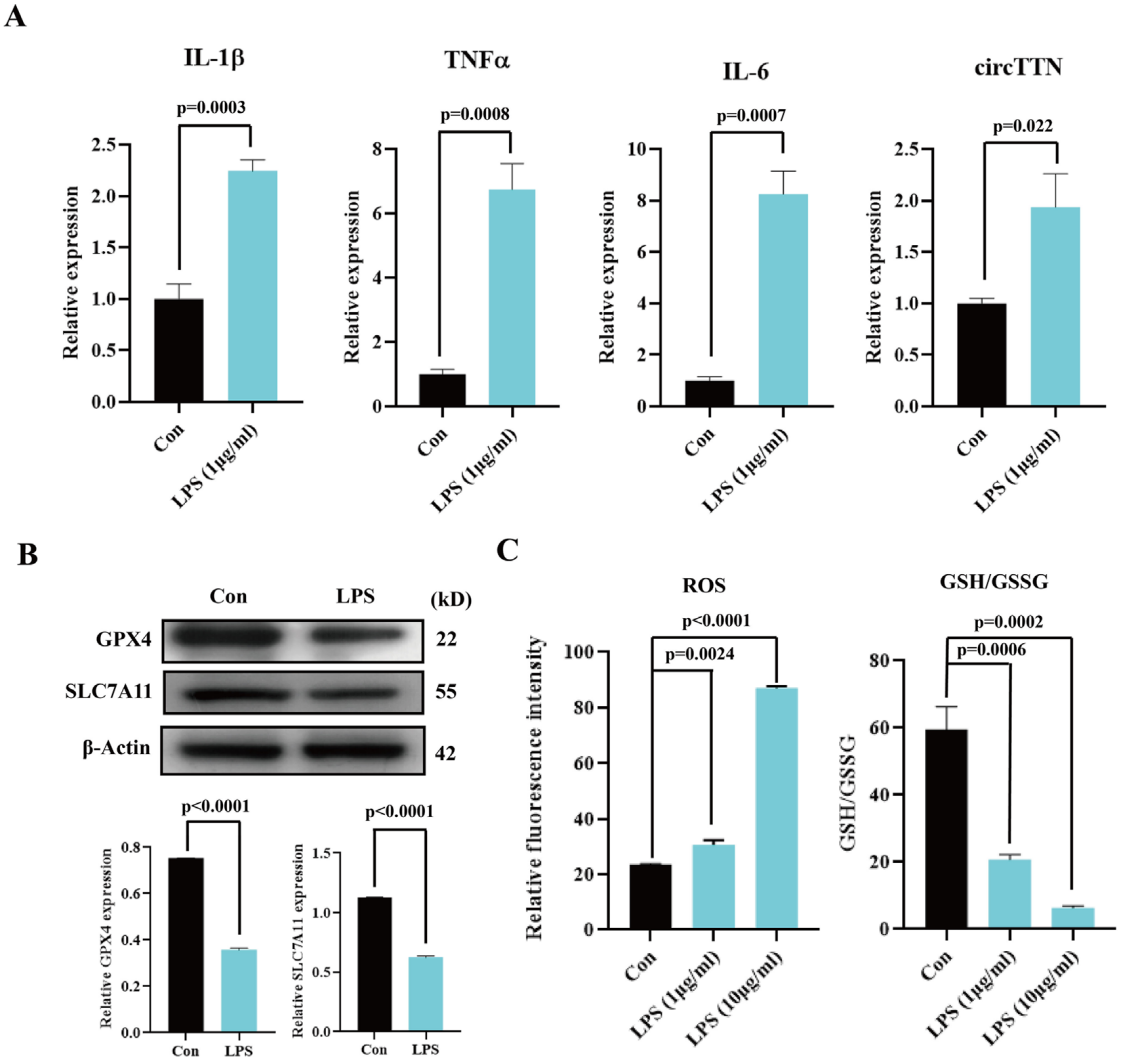
Ferroptosis in LPS‐treated HL‐1 cells. (A) qPCR detection of inflammatory factor genes in LPS‐treated HL‐1; (B) Western blot detection of the expression of ferroptosis regulator genes in LPS‐treated HL‐1 cells; (C) ROS and GSH detection in LPS‐treated HL‐1. Three biological repeats were performed for all qPCR and biochemical detections. Student *t*‐test was used for two‐group comparison, *p < 0.05, **p < 0.05, ***p < 0.001.

Results revealed a marked increase in the production of pro‐inflammatory cytokines such as interleukin‐1β (IL‐1β), tumour necrosis factor‐alpha (TNF‐α), and interleukin‐6 (IL‐6) in HL‐1 cells exposed to LPS (Figure 2A). Meanwhile, qPCR results showed that circTTN was increased in the LPS‐treated group compared to the control group (Figure 2A).

### 3.3 LPS Promotes Ferroptosis in the SCM Cell Model

Ferroptosis is a recently reported programmed cell death pathway that is caused by increased ROS and lipid peroxidation [18]. Although LPS‐treated HL‐1 cells showed increased ROS levels, whether this leads to ferroptosis remains unclear [18]. In this way, we assessed ferroptosis in the LPS‐treated HL‐1 model. As expected, LPS‐treated HL‐1 cells exhibited characteristics of ferroptosis (Figure 2B). As a quality control parameter, ROS and GSH measurements showed that after LPS treatment, GSH levels were significantly decreased while ROS levels were significantly increased in HL‐1 cells (Figure 2C).

### 3.4 Modulating circTTN Expression Affects the Role of LPS in the SCM Cell Model

**FIGURE 3:**
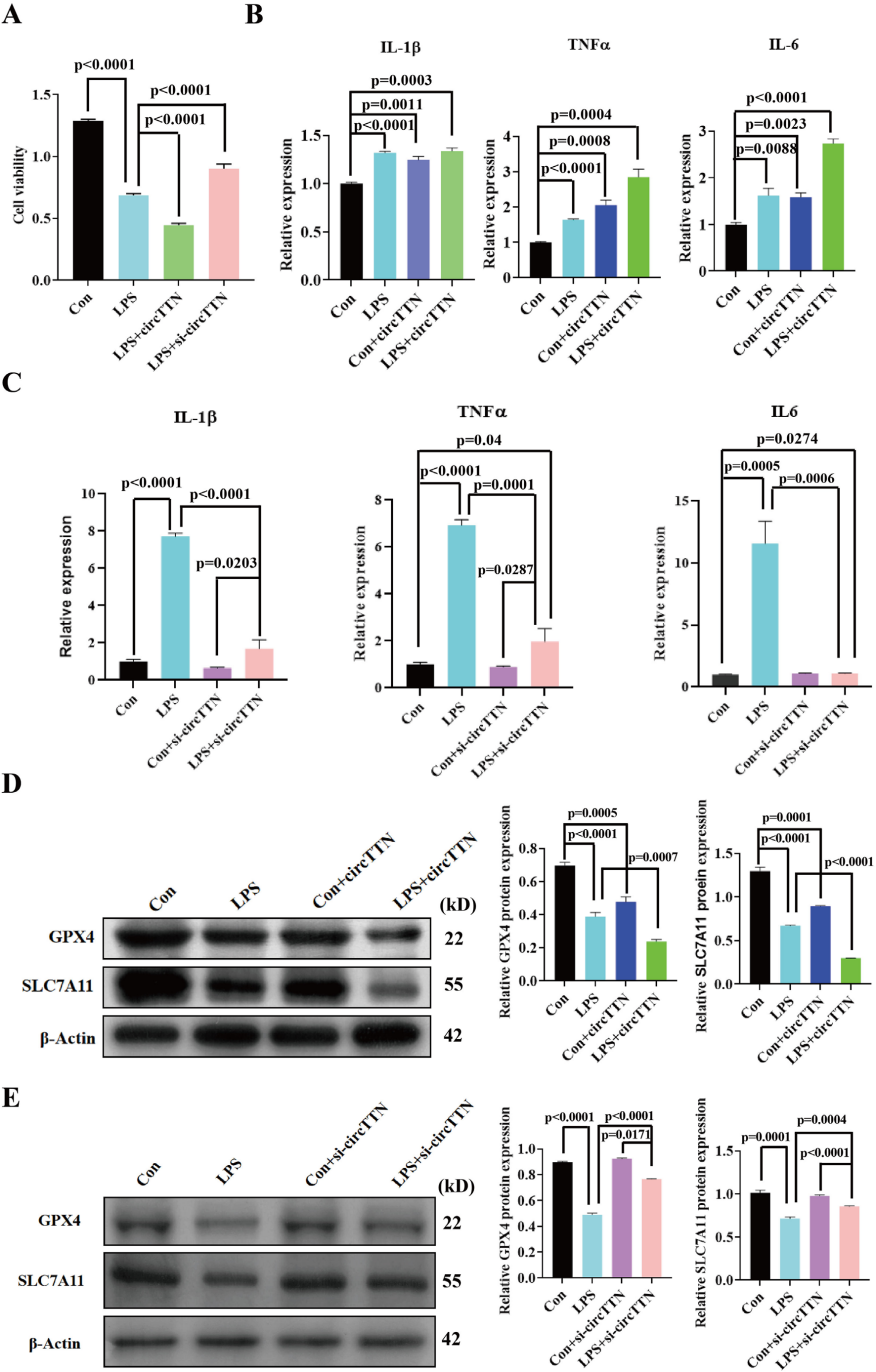
Expression of circTTN influences the ferroptosis sensitivity in LPS‐treated HL‐1 cells. (A) CCK8 detection after over‐expression or knockdown of circTTN; (B, C) qPCR detection of inflammatory factor genes after over‐expression or knockdown of circTTN; (D, E) Western blot detection of the expression of ferroptosis regulator genes after over‐expression or knockdown of circTTN. Three biological repeats were performed for all qPCR detections. Student's *t*‐test was used for two‐group comparison, *p < 0.05, **p < 0.05, ***p < 0.001.

Furthermore, to explore the effect of intervention circTTN expression on the role of LPS in HL‐1, we constructed a system of circTTN overexpression and interference, and analysed the changes of cell proliferation, inflammatory factor expression, and ferroptosis before and after intervention. As expected, LPS treatment led to growth inhibition (Figure 3A) and increased expression of TNF‐α, IL‐1β, and IL‐6 (Figure 3B). Overexpression of circTTN further inhibited cell viability (Figure 3A) and increased expression of TNF‐α, IL‐1β, and IL‐6 (Figure 3B). Knockdown of circTTN by siRNA promoted cell growth (Figure 3A) and decreased expression of TNF‐α, IL‐1β, and IL‐6 (Figure 3C). Expression of GPX4 and SLC7A11 was decreased after overexpression of circTTN (Figure 3D), whereas si‐circTTN led to increased expression of GPX4 and SLC7A11 after LPS treatment (Figure 3E).

### 3.5 circTTN Knockdown Weakened the Effects of SCM in Mouse and Cell Model

**FIGURE 4:**
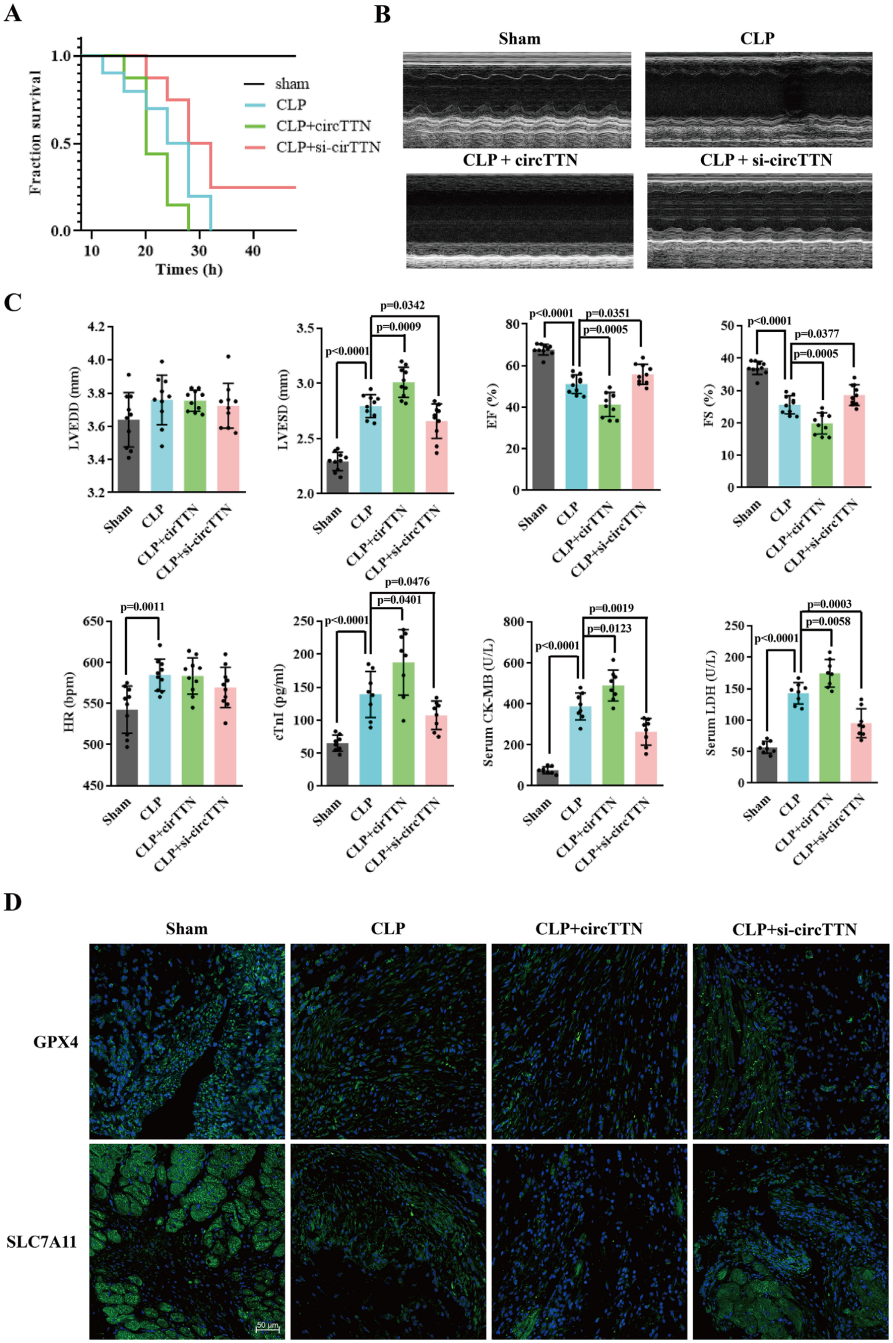
Effects of circTTN in CLP‐induced SCM model. (A) The survival states in the 10 CLP‐induced sepsis model; (B) Ultrasound detection of 10 mouse hearts in the CLP‐induced sepsis model; (C) ELISA detection of serum biochemical indices in the 8 CLP‐induced sepsis model; (D) Immunofluorescence for ferroptosis genes in the 8 CLP‐induced sepsis model. Student's *t*‐test was used for two‐group comparison, *p < 0.05, **p < 0.05, ***p < 0.001.

To analyse the effects of circTTN in a mouse septic model, a CLP‐induced mouse sepsis model was constructed as reported. Ultrasound detection showed that several cardiac physiological indexes were rescued in the si‐circTTN group (Figure 4A). The survival state was also rescued in the si‐circTTN group (Figure 4B). Several physiological and serological indicators showed that si‐circTTN ameliorated the adverse effects in the CLP‐induced SCM model (Figure 4C). Detection of the expression of ferroptosis regulators, GPX4 and SLC7A11, in CLP models after overexpression or knockdown of circTTN showed that the expression of GPX4 and SLC7A11 was decreased in CLP models compared to the sham group (Figure 4D). Overexpression of circTTN led to a further decrease of GPX4 and SLC7A11 compared to the CLP group. Whereas the expression of GPX4 and SLC7A11 was increased compared to the CLP group following the knockdown of circTTN by siRNA.

### 3.6 circTTN Binds With HSPA5 and Promotes Ferroptosis

**FIGURE 5:**
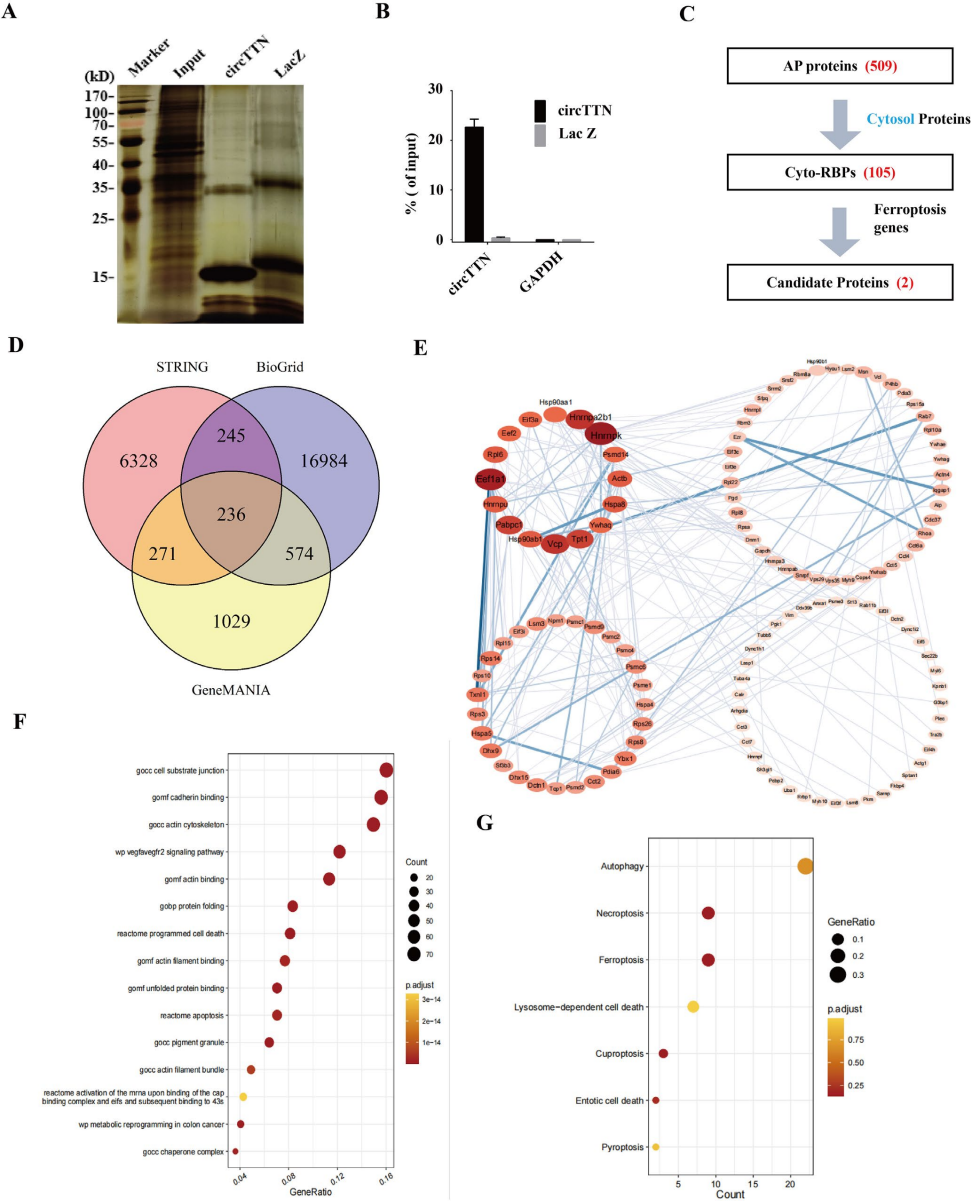
circTTN interaction proteins in HL‐1 cells. (A) RNA pull‐down capture the interaction proteins in HL‐1 cells, PCR detection circTTN in corresponding groups. Silver staining for the captured proteins; (B) qPCR detection of circTTN in corresponding groups; (C) 509 captured proteins, containing 105 Cyto‐RBPs, 2 ferroptosis regulate protein; (D) PPI analysis in STRING, BioGrid and GeneMANIA database; (E) PPI graph of captured proteins; (F) MsigDb pathway assay for captured proteins; (G) Programed cell death pathway assay for captured proteins.

To explore the mechanism of circTTN‐mediated ferroptosis, we used RNA pull‐down to capture the interacting proteins with circTTN (Figure 5A). QPCRs detecting the circTTN in RNA pull‐down samples showed that circTTN was significantly enriched in the circTTN captured sample (Figure 5B). In summary, 509 proteins were captured; among them, 105 RNA‐binding proteins that localised in the cytoplasm, 2 of which are reported ferroptosis regulatory proteins (Figure 5C). All captured proteins were subjected to PPI analysis using the STRING, BioGrid, and GeneMANIA databases (Figure 5D,E). MsigDb pathway assay (Figure 5F) and programmed cell death pathway analysis (Figure 5G) of the captured proteins are shown. The ferroptosis pathway was significantly enriched (Figure 5G).

**FIGURE 6:**
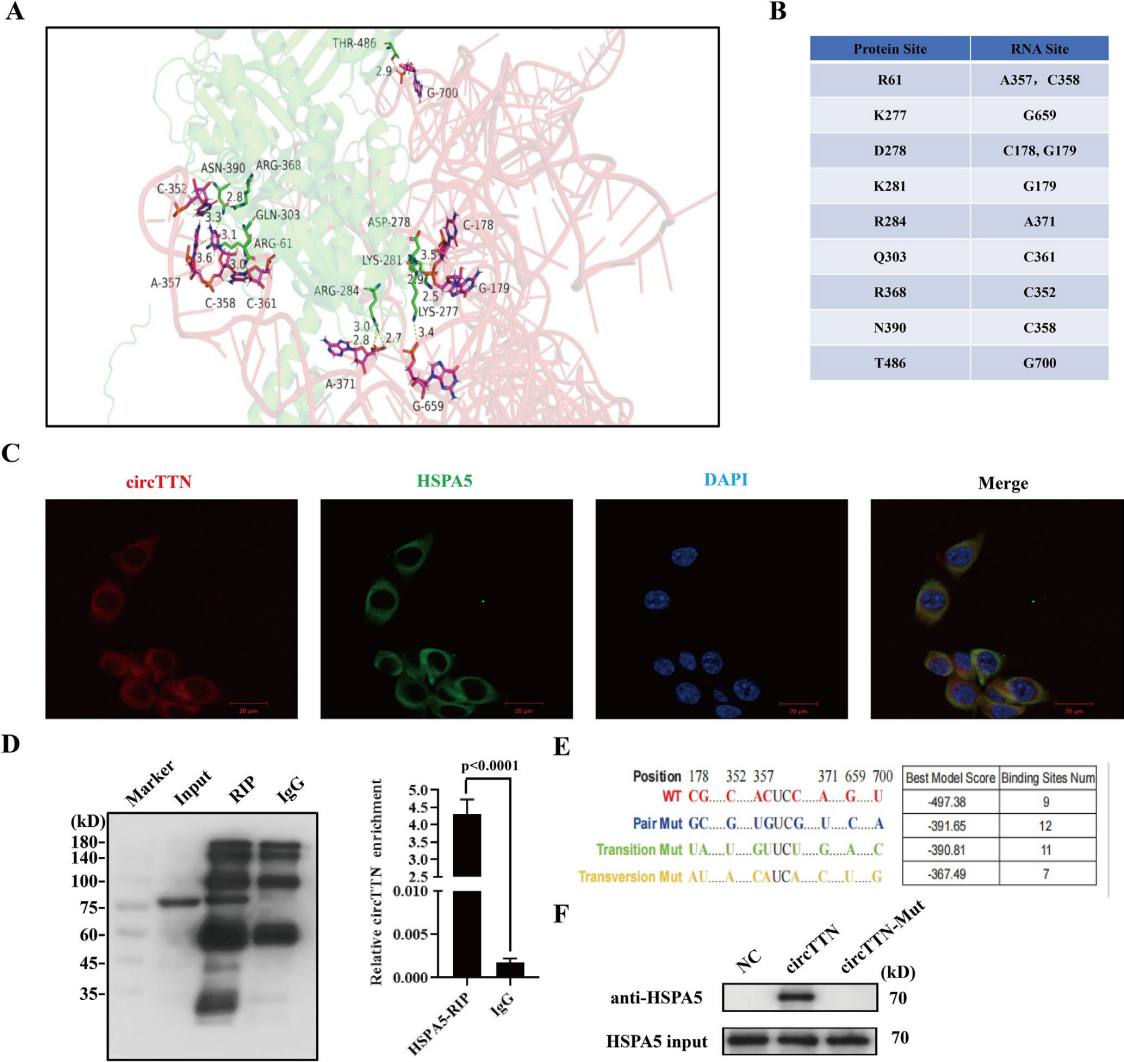
circTTN interacts with HSPA5. (A) Docking assay of the interaction of circTTN with HSPA5; (B) The binding site between HSPA5 and circTTN; (C) Immunofluorescence and FISH colocalisation analysis for HSPA5 and circTTN in HL‐1; (D) RIP‐qPCR detection of HSPA5 and circTTN; (E) Western blot detection of HSPA5 in RNA pull‐down of wild type (WT) and mutated (Mut) circTTN overexpressed HL‐1 cell samples. Three biological repeats were performed for all qPCR detections. Student's *t*‐test was used for two‐group comparison, *p < 0.05, **p < 0.05, ***p < 0.001.

The interaction between circTTN and heat shock protein 70 kDa protein 5 (HSPA5) was analysed by docking assay (Figure 6A). The binding sites for the interaction between circTTN and HSPA5 are shown in Figure 6B. To further validate the interaction between circTTN and HSPA5, we first detected the co‐localisation of circTTN and HSPA5 in cells by immunofluorescence and FISH co‐staining. The results showed that circTTN and HSPA5 co‐localised in the cytoplasm (Figure 6C). Anti‐HSPA5 antibody was used for RIP precipitation capture, and qPCR was used to detect the captured molecules. The results showed that circTTN could be co‐captured by anti‐HSPA5 antibody, indicating that circTTN and HSPA5 interacted (Figure 6D). According to the interaction sites between circTTN and HSPA5, we constructed the mutation of circTTN (circTTN‐mut). RNA pull‐down was used to analyse the interaction of circTTN or circTTN‐mut with HSPA5 protein. Western blot for the antisense probe captured proteins of circTTN or circTTN‐mut overexpressed HL‐1 cells, results showed that wild‐type circTTN could capture the HSPA5 protein, while mutated circTTN could not (Figure 6E).

## 4 Discussion

Sepsis represents a critical systemic condition that arises from an aberrant host response to infection [38]. A notable complication associated with sepsis is septic cardiomyopathy, which substantially impairs cardiac function and may result in grave outcomes [15]. In recent years, advancements in molecular biology have led to an increasing recognition of the significance of circRNA and ferroptosis in the onset and progression of various diseases [17, 36, 39, 40, 41, 42]. Several circRNAs have been identified to play roles in septic cardiomyopathy [43, 44]. It has been reported that some circRNAs influence the inflammatory response and myocardial damage in septic cardiomyopathy by regulating the expression of inflammatory factors [45, 46]. These circRNAs may also affect the death and survival of cardiomyocytes by regulating signalling pathways associated with oxidative stress and cell death [47, 48, 49]. The TTN gene is located at the q31 segment of chromosome 2 in humans and contains 364 exons, and extensive mRNA splicing yields a variety of isoforms [50]. Mutations of the TTN gene usually lead to dilated cardiomyopathy (DCM) [51, 52, 53]. Circular RNA is a new type of RNA splicing product that is manifested as a closed circle formation [24]. The function of circRNAs in human diseases has been widely reported [33, 36]. However, reports about the function of circRNAs in ferroptosis in sepsis cardiomyopathy are rare. Shuo Miao et al. reported circPIK3C2A competitive binds miR‐31‐5p and promotes expression of TFRC, leading to ferroptosis and accelerating myocardial injury [54]. Here, we found that circTTN was increased in the SCM animal model and cell model. Knockdown of circTTN not only inhibited SCM‐related ferroptosis and inflammatory factors, but also improved the survival rate, physiological and serological indicators of SCM. On the other hand, overexpression of circTTN in HL‐1 cells increased the expression of ferroptosis proteins and inflammation‐related immune factors. These data support that circTTN is a functional molecule in the ferroptosis pathway in both animal and cellular models of SCM.

The mechanism of circRNA function is mostly focused on the interaction with other biological molecules, such as miRNAs and proteins [33]. Whether circTTN binds with proteins is still unknown. circTTN is mostly located in the cytoplasm, which has been verified in Figure 1D,E. To find the functional mechanism of circTTN in regulating ferroptosis in SCM, we analysed the interacting proteins by RNA pull‐down assay. HSPA5, one of the proteins interacting with circTTN, is reported as a regulator of ferroptosis and is usually located in the cytoplasm [55]. Emerging evidence suggests a tight coupling between HSPA5 and ferroptosis, revealing a novel dimension to its biological functions [56, 57, 58, 59]. HSPA5, as a master regulator of ER homeostasis, senses and responds to perturbations in cellular environments, including oxidative stress—a hallmark of ferroptosis [55, 60, 61, 62]. HSPA5 can directly or indirectly influence the production and scavenging of reactive oxygen species (ROS) and the regulation of lipid peroxidation [63, 64]. Mechanisms of HSPA5 in regulating ferroptosis include: Modulation of antioxidant defence systems [60, 62, 65]; interacting and regulating some ferroptosis‐inducing proteins, like ACSL4 and FASN [66]; regulation of iron homeostasis [55, 67]. HSPA5 can also function by interacting with non‐coding RNAs [68, 69], including circRNAs [70]. This functional approach greatly expands our understanding of the mechanism by which HSPA5 functions. Instances of HSPA5 directly interacting with circRNAs are rarely reported. In this study, we identified that circTTN can bind to HSPA5, thereby influencing the susceptibility of cells to LPS‐induced ferroptosis. Additionally, we conducted a docking assay to investigate the binding site of circTTN with the HSPA5 protein, resulting in the identification of base sites within circTTN and corresponding amino acid sites in HSPA5. Upon mutating the base sites within circTTN, the interaction between circTTN and HSPA5 was disrupted. The clinical use of circTTN as a predictive and diagnostic marker, as well as a therapeutic target or drug for SCM, is made possible by the clarification of the mechanism of interaction between circTTN and HSPA5 [71, 72].

However, we only conducted studies about circTTN in the myocardial tissues and cells of septic models. A large and abundant protein in striated muscle is encoded by the TTN gene. Nevertheless, the circTTN levels in blood and other types of muscles in septic models have not yet been identified. Sepsis can result in multiple organ dysfunction, including septic encephalopathy, in addition to cardiomyopathy. It is unclear if circTTN has a comparable function in other organ dysfunction or tissue injury in septic models, which could be the focus of our upcoming study.

In conclusion, our investigation has demonstrated that circTTN serves as a significant factor in both animal and cellular models of SCM, where it enhances ferroptosis in cardiomyocytes. The downregulation of circTTN leads to a decreased susceptibility of cardiomyocytes to ferroptosis in SCM, thereby mitigating the clinical manifestations of SCM and enhancing the overall prognosis. This indicates that circTTN may represent a promising novel therapeutic target for SCM. Furthermore, circTTN has been shown to interact directly with HSPA5, effectively inhibiting its role in mediating ferroptosis. These findings provide preliminary insights into the expression and functionality of circTTN in the context of SCM, as well as initial validation of the effects of circTTN suppression in both in vitro and in vivo mouse models. This lays a foundational basis for the potential development of circTTN‐centred therapeutic strategies for SCM. Nonetheless, it is important to note that this research is primarily based on mouse and cellular models, necessitating further clinical validation of circTTN expression and its prospective therapeutic implications in future studies.

## Author Contributions

**Tingting Zhang:** conceptualization, formal analysis, validation, visualization, resources, project administration. **Danyang Zhao:** data curation, investigation, formal analysis, methodology, writing – original draft, project administration. **Qian Lei:** data curation, investigation, formal analysis, methodology, supervision, writing – original draft, writing – review and editing, funding acquisition. **Kunjiao Xue:** data curation, investigation, project administration, writing – review and editing, software. **Zhen Wei:** conceptualization, data curation, validation, visualization, writing – original draft, project administration. **Juanjuan Lyu:** funding acquisition, conceptualization, methodology, project administration, supervision, writing – review and editing. **Xue Li:** conceptualization, data curation, investigation, methodology, resources, writing – review and editing.

## Funding

This work was supported by the National Natural Science Foundation of China (82470290 and 82572491), the Fundamental Research Funds for the Central Universities (SCU2023G4175) and the Clinical Research Program of the People's Republic of China (WKZX2023CX170004).

## Ethics Statement

Animal experiments were approved by the Animal Care and Use Committee at Sichuan Provincial People's Hospital (Approval No. 2023–163).

## Conflicts of Interest

The authors declare no conflicts of interest.

## Data Availability

The data that support the findings of this study are available from the corresponding author upon reasonable request.
